# Successful Treatment of Acanthamoeba Keratitis According to New Protocol with Polihexanide 0.08% Therapy: Case Report

**DOI:** 10.3390/reports8020044

**Published:** 2025-04-04

**Authors:** Tomislav Kuzman, Suzana Matić, Ivan Gabrić, Antonela Geber, Ana Meter

**Affiliations:** 1 Department of Ophthalmology, School of Medicine, University Hospital Centre Zagreb, 10000 Zagreb, Croatia; tomislav.kuzman@kbc-zagreb.hr; 2 Department of Ophthalmology, University Hospital Centre Osijek, 31000 Osijek, Croatia; suzimatic72@gmail.com; 3 University Eye Clinic Svjetlost, 10000 Zagreb, Croatia; ivan.gabric@svjetlost.hr; 4 Department of Ophthalmology, Dubrava University Hospital, 10000 Zagreb, Croatia

**Keywords:** corneal infection, contact lens-related keratitis, Acanthamoeba keratitis, polyhexanide, case report

## Abstract

**Background and Clinical Significance:** Acanthamoeba keratitis (AK) is a rare but serious corneal infection that can lead to severe visual impairment or blindness if not promptly treated. The condition is primarily associated with contact lens use but can also occur due to ocular trauma or environmental contamination. The most frequently used treatment options include biguanides and diamidines, though dosing protocols remain empirical and vary widely among clinicians. Recent research has explored a new standardized protocol with 0.08% polihexanide (polyhexamethylene biguanide, PHMB) as a monotherapy for AK, offering improved efficacy and better corneal penetration. **Case Presentation**: This case report describes a 35-year-old female contact lens wearer who presented with redness, pain, photophobia, and vision loss in her right eye. Upon referral, a slit-lamp examination revealed stromal infiltrates and perineural involvement, with in vivo confocal microscopy (IVCM) confirming Acanthamoeba cysts. The patient was treated with a new standardized intensive regimen of polihexanide 0.08% monotherapy, leading to rapid clinical improvement. Corneal infiltrates were significantly reduced, and the best-corrected visual acuity (BCVA) improved from 0.4 logMAR to 0.15 logMAR. Resolution with only discrete stromal haze was achieved over the following months, without recurrence. **Conclusions:** This case highlights the potential of polihexanide 0.08% monotherapy as an effective treatment for AK in a new standardized treatment protocol.

## 1. Introduction and Clinical Significance

The Acanthamoeba keratitis (AK) is a rare but severe corneal infection that can cause intense pain, corneal perforation, or scarring and may ultimately lead to severe visual impairment or even blindness if left untreated. It is caused by Acanthamoeba, an opportunistic, free-living protozoan. Their life cycle includes trophozoite and cyst stages. The trophozoite is amoeboid with pseudopodia, while the cyst is a dormant form. Several species of Acanthamoeba are known, with A. castellanii and A. polyphaga being the most frequently associated with keratitis [1,2,3]. In addition to keratitis, Acanthamoeba can cause granulomatous amoebic encephalitis (GAE), disseminated acanthamoebiasis, and infections of the skin and sinuses [4,5]. Unlike systemic disease, AK can affect otherwise healthy, often young individuals. This is attributed to the fact that contact lens use is the key risk factor. Other contributing factors include eye trauma, contamination with water or soil, and chronic ocular disease [1,3,6].

Symptoms include pain, tearing, and photophobia, which are most often unilateral but can also be bilateral, especially in contact lens users. The pain can be severe, even in the early stages of AK, and may appear disproportionate to the clinical findings. However, some patients experience no pain, and its absence does not exclude AK from the differential diagnosis [1,7].

In the initial stages of AK, clinical signs vary and may include punctate keratopathy, pseudodendrites, and epithelial or subepithelial infiltrates. Perineural infiltrates are pathognomonic for AK and are associated with the neurotropism of *Acanthamoeba* trophozoites. Limbitis can occur in both early and late stages. As the disease progresses, stromal involvement develops, often with a characteristic ring-shaped or multifocal stromal infiltrate. In the advanced stages, ulceration, anterior uveitis, hypopyon, and corneal edema may also occur [4,8].

AK is often misdiagnosed. In its early stages, it can be mistaken for viral keratitis due to pseudodendritic lesions, while in later stages, it may be misidentified as bacterial or fungal keratitis due to the presence of stromal ring infiltrates. Initially, the disease progresses slowly, but in later stages, it can advance more rapidly [1,4].

The diagnosis of AK is based on a combination of clinical suspicion, microbiological analysis (smears and cultures), polymerase chain reaction (PCR) and in vivo confocal microscopy (IVCM) [4,9]. IVCM is a noninvasive technique for corneal imaging. It enables visualization of *Acanthamoeba* cysts, which appear as hyperreflective, double-walled, spherical structures (15–30 µm) located in the epithelium or stroma. Hyperreflective trophozoites, which are significantly larger, are rarely observed [4,10]. The density of cysts and the depth of infiltration have been associated with disease severity [9,11,12,13].

The most frequently used drugs effective against both trophozoites and more resilient cysts are biguanides, either as monotherapy or in combination with diamidines. Biguanides include chlorhexidine and polyhexamethylene biguanide (PHMB), while diamidines include propamidine isethionate and hexamidine-diisethionate [14,15]. Other investigated treatments, such as voriconazole, oral miltefosine, and photochemical therapy with PACK-CXL, have limited supporting evidence in the literature and require further research [14,15,16]. Therapeutic keratoplasty has been associated with a relatively high risk of graft failure and poor visual outcomes [15,17,18].

## 2. Case Presentation

**Figure 1:**
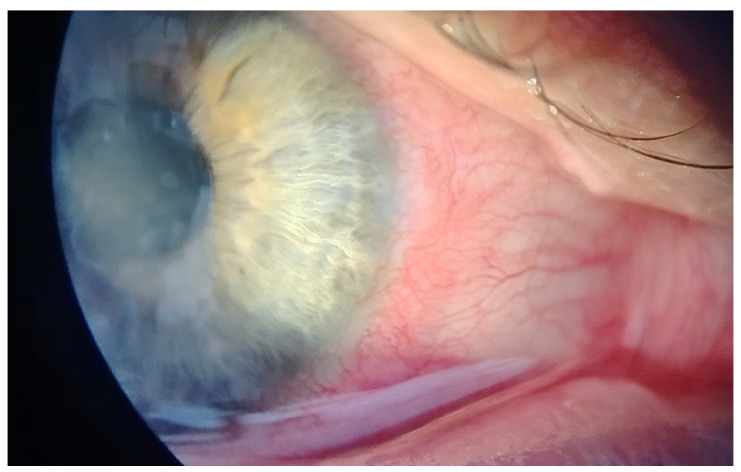
Perilimbal injection.

**Figure 2:**
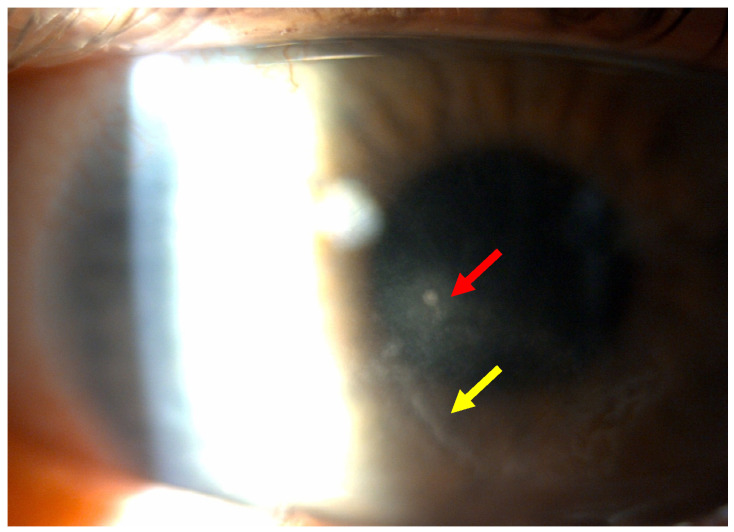
Stromal infiltrate (red arrow) and ring-shaped infiltrate (yellow arrow).

**Figure 3:**
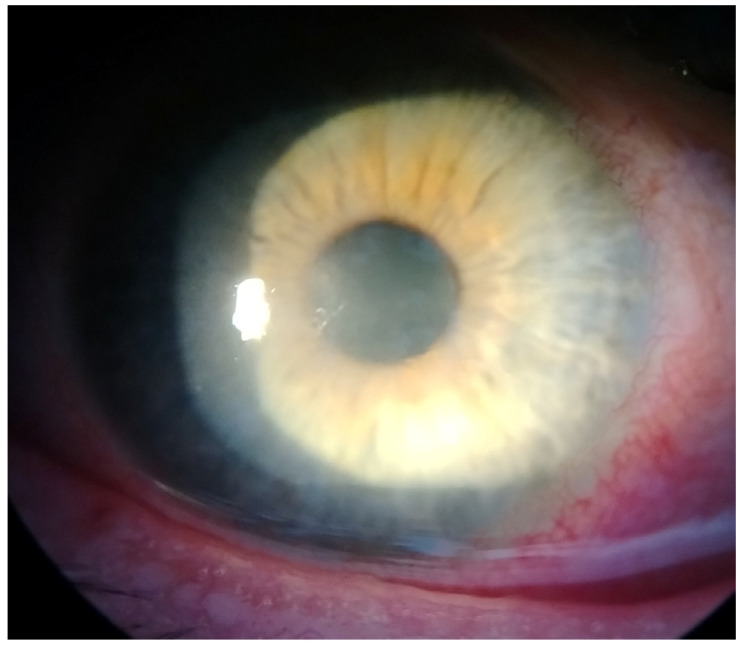
Perineural infiltrates.

This case report describes a 35-year-old female who presented with redness, pain, tearing, photophobia, and vision loss in her right eye, beginning in mid-September 2024. The patient was a soft contact lens wearer due to myopia of −6.00 DS and reported showering and occasionally sleeping while wearing her contact lenses. She was initially treated for five days with topical corticosteroids and antibiotics by her primary care doctor, with no improvement, after which she was referred to an ophthalmologist. At her initial presentation in October 2024 at the University Hospital Centre Zagreb, the best-corrected visual acuity (BCVA) of the right eye was 0.4 logMAR. Slit-lamp examination revealed pronounced perilimbal injection (Figure 1), two fluorescein-negative stromal infiltrates at the 4 and 8 o’clock positions, and diffusely reduced corneal transparency with a centrally located ring-shaped infiltrate (Figure 2). Perineural infiltrates were also observed (Figure 3).

The anterior chamber was clear, with no pathological findings. Additionally, corneal neovascularization was observed in the superior and inferior poles of the cornea in both eyes.

**Figure 4:**
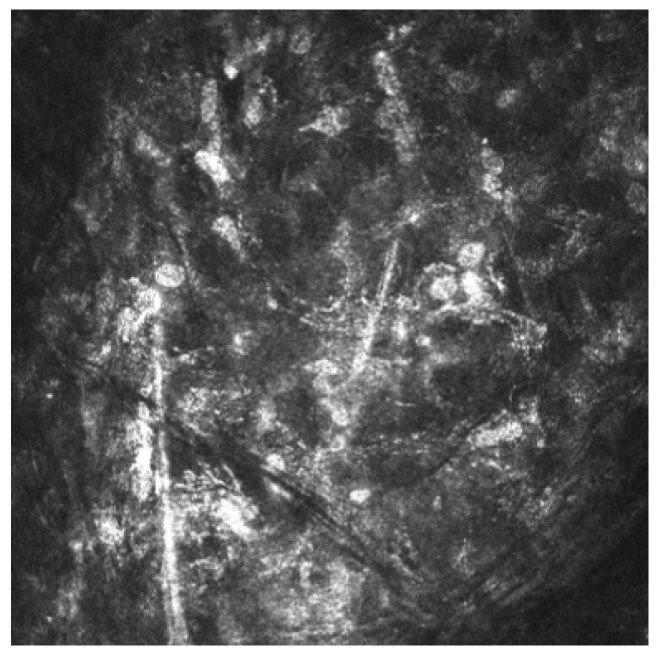
Acanthamoeba cysts in corneal in vivo microscopy.

Acanthamoeba keratitis was confirmed by in vivo corneal microscopy (Figure 4).

The patient discontinued contact lens wearing and was treated according to a new standardized protocol described in the ODAK trial (Table 1), which consisted of polihexanide 0.08% administered every hour while awake (16 times per day) for five days, with no overnight treatment [19]. Subsequent treatment involved administration every two hours (eight times per day) for seven days, followed by every three hours (six times per day) for another seven days, with no overnight treatment during any phase of therapy.

There were no allergic reactions or adverse effects observed following the administration of treatment. After this 19-day intensive course of anti-amoebic therapy, the patient’s BCVA in the right eye improved to 0.3 logMAR, with a cleared fluorescein-negative cornea and two reduced stromal infiltrates. Finally, the patient was treated every four hours (four times per day) for an additional month, after which there were no signs of recurrence and the treatment was discontinued at a final visual acuity of 0.15 logMAR.

**Figure 5:**
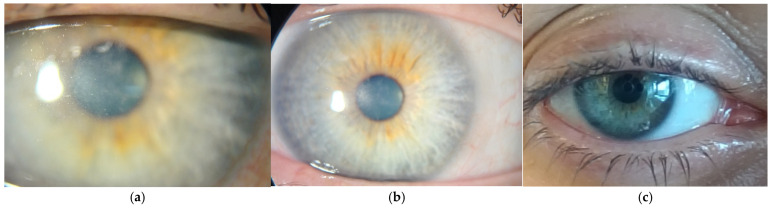
(**a**) Forty days after starting polihexadine 0.08% protocol. (**b**) Sixty days after starting polihexadine 0.08% protocol. (**c**) Ninety days after starting polihexadine 0.08% protocol.

There were several follow-up examinations conducted at 40 (Figure 5a), 60 (Figure 5b), and 90 (Figure 5c) days after the initiation of treatment, all of which showed further improvement in corneal transparency and some remaining perineural infiltrates.

Control IVCM performed 90 days after the initiation of treatment did not find any Acanthamoeba cysts.

Throughout the follow-up period, neither topical nor systemic steroids or NSAIDs were administered. No additional treatments were required during the extended follow-up period.

## 3. Discussion

Acanthamoeba keratitis (AK) remains a challenging condition to diagnose and manage. Early and accurate identification of the pathogen is critical for initiating effective therapy and preventing irreversible corneal damage.

In a national survey by Jasim et al., 25% of Acanthamoeba keratitis cases were diagnosed based solely on clinical presentation due to the high false-negative rates of other diagnostic methods [20]. In vivo confocal microscopy (IVCM) is a noninvasive imaging method that has been shown in the literature to have a higher sensitivity than PCR and culture in the diagnosis of Acanthamoeba keratitis [21].

The new standardized treatment regimen with topical polihexanide 0.08% (PHMB) monotherapy demonstrated rapid and effective clinical improvement in the patient presented in this case report. After 90 days of intensive treatment, corneal infiltrates were significantly reduced, and the patient’s BCVA improved from 0.4 logMAR to 0.15 logMAR. Resolution was achieved without recurrence over the following months, with only discrete remaining stromal haze.

In current clinical practice, AK is most commonly treated with a combination of biguanides (PHMB 0.02% or chlorhexidine 0.02–0.06%) and diamidines (propamidine 0.1%). However, these treatments are often based on clinical experience rather than standardized protocols, leading to variations in dosing and approach among clinicians and institutions [14,15,22].

Recent literature has explored PHMB monotherapy as a potential primary treatment for AK due to its low toxicity and high efficacy against both trophozoites and cysts [1,14,19,22,23].

Papa et al. (2019) provided evidence that PHMB 0.02% monotherapy is as effective as other widely used anti-amoebic treatments, supporting its use as a first-line therapy for AK due to its simplicity and lower cost compared to combination therapy with diamidines [14].

More recent findings from Papa et al. (2022) suggest that PHMB 0.08% may be a well-tolerated option for treating deep stromal invasion in AK, offering higher drug concentrations at the site of infection [23].

The new standardized protocol from the ODAK trial by Dart et al., based on polihexanide 0.08% monotherapy, as applied in this case study, represents a promising advancement in AK treatment [19]. A preliminary report of two cases by Di Zazzo et al. showed a strong therapeutic response to polihexanide 0.08%, significantly reducing illness duration and lowering the risk of recurrence [24].

Although this is a single case report without a control group, the observed rapid clinical improvement following polihexanide 0.08% monotherapy is consistent with findings from larger studies [19,23], supporting its efficacy and tolerability in AK treatment. Despite these promising results, further large-scale studies and randomized controlled trials are necessary to establish polihexanide 0.08% as a definitive first-line treatment.

While no recurrence was observed within the six-month follow-up period, the limited duration restricts conclusions regarding long-term outcomes. Long-term follow-up is essential to assess the risk of recurrence and potential late complications. Future investigations with extended observation periods are necessary to provide further insight into long-term outcomes.

In this case, treatment was administered according to the standardized protocol developed in the ODAK trial, which includes a defined dosing schedule and clear criteria for tapering and discontinuation. The application of this protocol has been associated with favorable clinical outcomes and a reduced duration of treatment [19,24], as also observed in the present case report. Further efforts to refine and validate standardized protocols through additional clinical studies may contribute to more consistent and effective care for patients with Acanthamoeba keratitis.

## 4. Conclusions

Acanthamoeba keratitis remains a challenging condition requiring early diagnosis and prompt treatment to prevent severe visual impairment. This case highlights the efficacy of polihexanide 0.08% monotherapy, showing rapid clinical improvement, reduced illness duration, and complete resolution without recurrence. Given its potential advantages over combination therapies, this novel standardized protocol with polihexanide 0.08% may represent a promising first-line treatment for AK.

## Figures and Tables

**Table 1:** Dosing regimen adapted from the ODAK study [19].

Days	Frequency	Number of Drops/Day
Day 0–5 (5 days)	Every hour during the day	16
Day 6–12 (7 days)	Every 2 h during the day	8
Day 13–19 (7 days)	Every 3 h during the day	6
Day 20 until the resolution	4 times daily	4

## Data Availability

The data supporting the findings of this study are not publicly available due to the presence of personal information and ethical considerations.

## Abbreviations

The following abbreviations are used in this manuscript:

AK	Acanthamoeba keratitis
GAE	Granulomatous amoebic encephalitis
IVCM	In vivo confocal microscopy
PHMB	Polyhexamethylene biguanide
PACK-CXL	Photoactivated chromophore for keratitis corneal cross-linking
BCVA	Best-corrected visual acuity
NSAIDs	Nonsteroidal anti-inflammatory drugs
ODAK	Orphan Drug for Acanthamoeba Keratitis
